# Supplementary material for: Pathogenic and Endosymbiotic Bacteria and Their Associated Antibiotic Resistance Biomarkers in Amblyomma and Hyalomma Ticks Infesting Nguni Cattle (Bos spp.)
Source: Pathogens. 2022 Apr 2;11(4):432. doi: 10.3390/pathogens11040432 (PMC9028808; doi:10.3390/pathogens11040432)
Supplement: Supplementary file 1 [file pathogens-11-00432-s001.zip › Supplementary Figure S1.pdf]

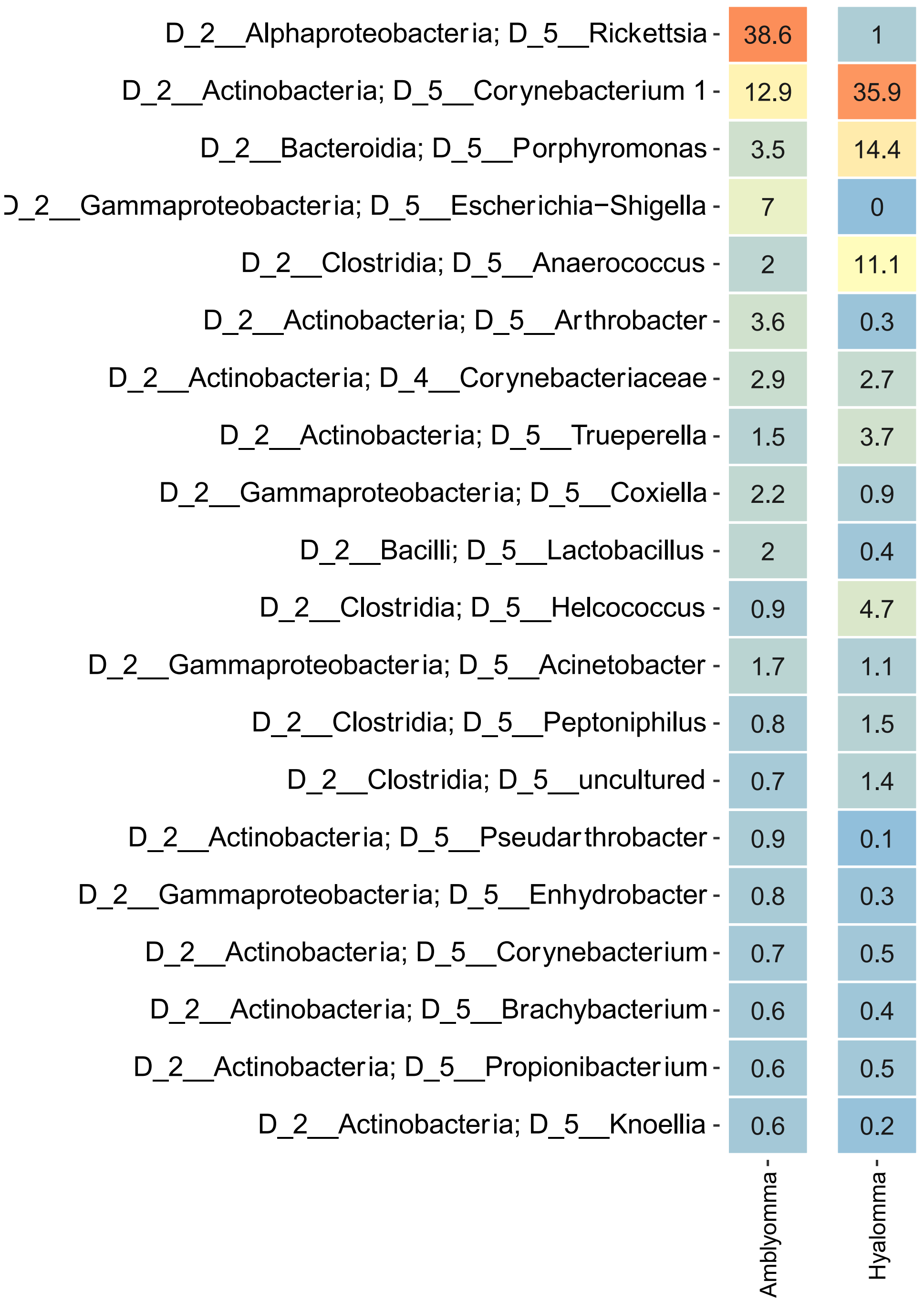

Figure S1. Comparative heatmap showing percentage grouped abundance of bacterial community at genus level
