# Supplementary material for: Pathogenic and Endosymbiotic Bacteria and Their Associated Antibiotic Resistance Biomarkers in Amblyomma and Hyalomma Ticks Infesting Nguni Cattle (Bos spp.)
Source: Pathogens. 2022 Apr 2;11(4):432. doi: 10.3390/pathogens11040432 (PMC9028808; doi:10.3390/pathogens11040432)
Supplement: Supplementary file 1 [file pathogens-11-00432-s001.zip › Supplementary Table S1.pdf]

Table S1. The core microbiome (unique and shared) associated with *Amblyomma* and *Hyalomma* ticks

OTUs	Taxonomy	Relative abundance (%)	*Amblyomma*	*Hyalomma*
Otu1085	Proteobacteria;Alphaproteobacteria;Rickettsiales;Rickettsiaceae;Rickettsia	42.64	+	-
Otu283	Actinobacteria;Actinobacteria_c;Corynebacteriales;Corynebacteriaceae;Corynebacterium	15.30	+	+
Otu139	Firmicutes;Tissierellia;Tissierellales;Peptoniphilaceae;Anaerococcus	3.39	-	+
Otu110	Firmicutes;Bacilli;Lactobacillales;Aerococcaceae;Aerococcus	1.44	-	+
Otu1185	Actinobacteria;Actinobacteria_c;Actinomycetales;Actinomycetaceae;Trueperella	1.43	-	+
Otu286	Proteobacteria;Gammaproteobacteria;Legionellales;Coxiellaceae;Coxiellaceae_uc	1.34	+	-
Otu647	Firmicutes;Tissierellia;Tissierellales;Peptoniphilaceae;Helcococcus	1.26	-	+
Otu1004	Firmicutes;Tissierellia;Tissierellales;Peptoniphilaceae;Peptoniphilus	0.77	-	+
Otu285	Proteobacteria;Gammaproteobacteria;Legionellales;Coxiellaceae;Coxiella	0.70	-	+
Otu1146	Firmicutes;Clostridia;Clostridiales;Ruminococcaceae;Sporobacter	0.56	-	+
Otu200	Actinobacteria;Actinobacteria_c;Micrococcales;Dermabacteraceae;Brachybacterium	0.55	-	+
Otu1030	Bacteroidetes;Bacteroidia;Bacteroidales;Porphyromonadaceae;Porphyromonadaceae_uc	0.54	-	+
Otu1089	Firmicutes;Clostridia;Clostridiales;Peptostreptococcaceae;Romboutsia	0.37	-	+
Otu689	Firmicutes;Clostridia;Clostridiales;Mogibacterium_f;JRNA_g	0.35	-	+
Otu1	Bacteroidetes;Bacteroidia;Bacteroidales;Bacteroidaceae;5-7N15_g	0.20	-	+
Otu225	Firmicutes;Tissierellia;Tissierellales;Peptoniphilaceae;CYUJ_g	0.17	-	+
Otu543	Firmicutes;Tissierellia;Tissierellales;Peptoniphilaceae;Finegoldia	0.16	-	+
Otu896	Firmicutes;Clostridia;Clostridiales;Christensenellaceae;PAC001207_g	0.15	-	+
Otu966	Bacteroidetes;Bacteroidia;Bacteroidales;AC160630_f;PAC002482_g	0.15	-	+
Otu618	Verrucomicrobia;Verrucomicrobiae;Verrucomicrobiales;Akkermansiaceae;HM630201_g	0.11	-	+
Otu1002	Firmicutes;Clostridia;Clostridiales;Peptococcaceae;Peptococcus	0.10	-	+
Otu473	Firmicutes;Clostridia;Clostridiales;Ruminococcaceae;Eubacterium_g23	0.09	-	+
Otu280	Actinobacteria;Coriobacteriia;Coriobacteriales;Coriobacteriaceae;Coriobacteriaceae_uc	0.09	-	+
Otu871	Firmicutes;Clostridia;Clostridiales;Ruminococcaceae;PAC000661_g	0.07	-	+
Otu1029	Proteobacteria;Alphaproteobacteria;Sphingomonadales;Erythrobacteraceae;Porphyrobacter	0.02	-	+

(+) represents the presents of the OTU while (-) represents no detection of the OTU
